# Integration of Machine Learning-Based Pathogenicity Prediction and Phenotype Matching Improves Variant Prioritization in Rare Clinical Testing

**DOI:** 10.3390/cimb48070706

**Published:** 2026-07-11

**Authors:** Jiri Ruzicka, Jean-Marie Ravel, Jérôme Audoux, Alexandre Boulat, Julien Thévenon, Kévin Yauy, Marine Dancer, Laure Raymond, Yannis Lombardi, Nicolas Philippe, Michael GB Blum, Nicolas Duforet-Frebourg, Laurent Mesnard

**Affiliations:** 1 SeqOne Genomics, 34000 Montpellier, France; jiri.ruzickajiri@seqone.com (J.R.); jerome.audoux@seqone.com (J.A.); alexandre.boulat@seqone.com (A.B.);; 2 Institute of Advanced Biosciences, CNRS UMR 5309, Université Grenoble Alpes, 38000 Grenoble, France; 3 LIRMM, CNRS, University of Montpellier, 34000 Montpellier, France; 4 Service de Génétique, Eurofins Biomnis, 69007 Lyon, France; 5 Service des Soins Intensifs Néphrologiques et Rein Aigu, Hôpital Tenon, Assistance Publique-Hôpitaux de Paris, 75020 Paris, France; 6 Centre Maladie Rare MAHREA and ERKNET, Hôpital Tenon, 75020 Paris, France; 7 INSERM CORAKID, Hopital Tenon, 75020 Paris, France

**Keywords:** variant prioritization, rare disease, machine learning, pathogenicity prediction, phenotype matching, ACMG, exome sequencing, clinical genomics, HPO, DiagAI

## Abstract

Genome and exome sequencing have become central to diagnosing rare hereditary diseases, but each test returns thousands of variants that a clinical scientist must review by hand to find the one responsible for the patient’s condition. This manual interpretation is the main bottleneck in clinical genomics. To reduce it, we developed DiagAI, a machine-learning system that ranks the variants found in a patient and returns a short list of the most likely causal candidates. DiagAI combines three sources of evidence: a pathogenicity score from the Universal Pathogenicity Predictor (UP2), a model we trained to estimate how damaging a variant is on the five-tier scale of the American College of Medical Genetics and Genomics (ACMG); a phenotype-matching score from PhenoGenius, which weighs how well a gene’s known clinical features match the patient’s symptoms (encoded as Human Phenotype Ontology, or HPO, terms); and expert rules covering inheritance pattern and sequencing quality. We evaluated DiagAI on 966 exomes from adults investigated for kidney disease of unknown cause, of which 196 had a confirmed genetic diagnosis. We first tested UP2 on its own by ranking 62 confirmed disease-causing missense variants that were absent from its training data: UP2 placed the causal variant within the top 100 candidates in 87% of cases, compared with 61% for the widely used tool REVEL. Across the 196 diagnosed exomes, the full DiagAI shortlist contained the causal variant in 94.9% of cases when the patient’s symptoms were provided and in 90.8% when they were not, with a typical shortlist of about 10 variants. When symptoms were provided, the single top-ranked variant was the correct diagnosis in 74% of cases, versus 42% without symptoms, exceeding the performance of the established tools Exomiser and AI-MARRVEL on the same cohort. DiagAI produces compact, accurate shortlists that can reduce the manual interpretation workload as diagnostic sequencing volumes continue to grow.

## 1. Introduction

Hereditary diseases are a significant health concern worldwide, and exome sequencing (ES) and whole-genome sequencing (WGS) have become essential for their diagnosis [1,2]. Efficient genomic variant interpretation is a critical step in clinical genomics [3].

To provide a molecular diagnosis, a large number of variants detected by high-throughput sequencing need to be interpreted. The American College of Medical Genetics and Genomics (ACMG) offers standardized guidelines for variant classification, grouping variants into five categories: pathogenic, likely pathogenic, uncertain significance, likely benign, and benign. Variants of uncertain significance are a major challenge [4] that mostly occur due to limited available data and insufficient prediction algorithms, although updated recommendations propose quantitative criteria for pathogenicity classification [5].

To address these challenges, artificial intelligence (AI) has emerged as a promising solution. Several studies have demonstrated AI’s potential to improve the efficiency of variant interpretation workflows, reduce analysis times, and alleviate the human workload [6,7,8,9] in order to facilitate access to personalized medicine.

We present DiagAI, a machine-learning system that ranks the variants detected in a patient and returns a short list of the most likely causal candidates. DiagAI draws on three complementary lines of evidence. First, it uses a molecular pathogenicity score from the Universal Pathogenicity Predictor (UP2), a classifier we developed that estimates how likely a variant is to be damaging, expressed on the five-tier ACMG scale. Second, when the patient’s clinical features are recorded as Human Phenotype Ontology (HPO) [10] terms, DiagAI incorporates a phenotype-matching score from PhenoGenius, a previously published tool that quantifies how well a gene’s known disease features match the patient’s symptoms and thereby upweights genes consistent with the reported phenotype. Third, DiagAI applies expert rules that check consistency with the expected mode of inheritance, including family transmission in sequenced trios, and that account for sequencing quality. By combining a learned pathogenicity model with phenotype matching and explicit expert rules, DiagAI is designed to reflect how a clinical scientist weighs evidence when interpreting a genome.

To evaluate DiagAI, we performed a retrospective analysis of 966 exomes from adults investigated for kidney disease of unknown cause, 196 of which had a confirmed genetic diagnosis. We benchmarked the system against established variant- and gene-prioritization tools, both for its molecular component (UP2 versus the missense predictors CADD, REVEL and AlphaMissense) and for the complete system (DiagAI versus the phenotype-aware tools Exomiser and AI-MARRVEL). DiagAI placed the causal variant within its shortlist in 94.9% of diagnosed cases when the patient’s symptoms were available and in 90.8% when they were not.

## 2. Materials and Methods

### 2.1. Study Design

To validate DiagAI, we conducted a retrospective analysis from exome sequencing (ES) data generated from adult participants (*n* = 966) with nephropathy of unknown origin, sequenced from March 2018 to July 2022 (Supplemental Table S1). Of these, 196 (24%) were considered positive cases, defined as containing a causal variant previously identified by a geneticist. The remaining 770 (76%) were considered negative cases, where no diagnosis could be established by a geneticist based on the exome sequencing data.

### 2.2. Exome Sequencing

DNA was extracted from peripheral blood using the QIAsymphony DSP DNA Mini Kit on a QIAsymphony instrument following the manufacturer’s (QIAGEN, Venlo, The Netherlands) guidelines. Library preparation and capture was performed with Twist reagents (Human Comprehensive Exome or Human Exome 2.0 Plus Comprehensive Exome Spike-in, Twist Bioscience, South San Francisco, CA, USA). Sequencing was performed on the Illumina NovaSeq6000 (Illumina, San Diego, CA, USA) in paired-end mode (2 × 150 bp reads). Raw data (bcl format) were converted to FASTQ format using BCL Convert v4.3.13. Reads were aligned to the human reference genome (UCSC Genome Browser build hg37) with Burrows–Wheeler Aligner [11] for maximal exact matches.

Calling was performed with an internal procedure, the GermlineVar pipeline, of SeqOne Genomics (Montpellier, France). The GermlineVar pipeline implements a comprehensive variant detection strategy utilizing multiple variant calling algorithms. The pipeline integrates Freebayes [12], GATK [13], GRIDSS [14], AluMEI (in-house pipeline [15]), and GATK-Mitochondrial pipeline [16] (versions ≥ 2.0). Detection sensitivity parameters are optimized according to the analysis type. In panel-based analyses, the Freebayes algorithm is configured to detect variants with allele frequencies ≥5%, while exome analysis maintains a more stringent threshold of ≥10% for SNVs. Notably, GRIDSS, AluMEI, and GATK operate independently of variant allele frequency thresholds for small variant detection, allowing for maximum sensitivity in structural variant identification.

### 2.3. Universal Pathogenicity Predictor (UP2)

The precise assessment of genomic variant pathogenicity is a fundamental prerequisite for the molecular diagnosis of rare diseases. UP2 is a machine learning model using the Gradient Boosting Decision Tree algorithm, implemented by the Python package XGBoost v3.3.0 [17], designed to predict the molecular pathogenicity of genomic variants. This prediction is based on 74 features derived from various evidence sources (see below) and linked to the ACMG criteria [18]. The classifier assigns a probability to each of the five ACMG classes for a given variant. These five scores are then aggregated into a final UP2 score.

The dataset used for training was extracted from ClinVar version 04-2024 [19] and transformed into a standard VCF file using the in-house open-source clinvcf package (https://github.com/SeqOne/clinvcf (accessed on 17 December 2024)) [20]. Variants with conflicting interpretations of pathogenicity were re-annotated with the removal of outlier submissions falling outside of the inter-quartile range [20]. Variants that stayed conflicting after the reannotation process were excluded. Only single-nucleotide variants (SNVs) and short indels were included. Biological annotations of variants were sourced from gnomAD v4.1 [21], VEP predictions version 107 [22] using RefSeq transcripts, dbNSFP [23] version 4.3, dbscSNV [24] version 1.1, CI-SpliceAI [25] v1.1.1, and the RMSK database [26] version 210903.

The dataset included 2.7 million variants from ClinVar [19], labeled according to the five ACMG pathogenicity categories [19]. It was split into a training set of 2.6 million variants—95% of the total dataset—to train the model using default hyperparameters, and a validation set of 130,000 variants—5% of the total dataset—to validate the model.

Data augmentation was then used to improve ClinVar data. Indeed, supplementary benign variants, either frequent or non-coding, were added into the dataset to better fit a real-world variant distribution.

### 2.4. UP2 Interpretability Features

To assess the relative importance of different ACMG criteria in our classification model, we calculated Shapley values for all 74 features used in the molecular pathogenicity score. These Shapley values were then aggregated in order to be mapped to the known ACMG criteria. This approach allowed us to quantify the contribution of each ACMG tag to the molecular UP2 score, providing insight into which criteria were most influential in determining variant pathogenicity according to our model. To calculate a Shapley value for each ACMG criterion, we summed the Shapley values of all features associated with that specific criterion.

### 2.5. DiagAI Prioritization Algorithm

The clinical interpretation of genomic data in rare diseases requires determining the pathogenicity of identified variants, establishing a link between the patient phenotypes and genes as well as a concordance between family transmission rules. DiagAI is a linear regression model, implemented by scikit-learn v1.4.1, trained to retrieve the most likely causal variant for each patient.

The prediction is based on (1) the molecular score UP2 detailed above; (2) a phenotypic score based on PhenoGenius algorithm [27] previously developed and available on GitHub (https://github.com/kyauy/PhenoGenius (accessed on 17 December 2024)); and (3) score adjustments based on features linked to inheritance pattern coherence and quality scores. The final score is between 0 and 100.

Phenotypic information was incorporated using HPO [10] terms, with gene prioritization performed by PhenoGenius, a tool that leverages gene-HPO term associations from literature-based matrices [27]. Inheritance mode consistency was sourced from PanelApp [28] version 240807, OMIM [29] version 240807, and MedGen [30] version 230331. Variant calling data (DP ≥ 5, AO ≥ 2, VAF ≥ 0.20), quality (base quality phred score ≥ 20, no PASS filter used), and parental variant data, in cases where trio sequencing was performed, were based on the GermlineVar pipeline detailed above.

DiagAI is organized as a two-layer architecture that mirrors the sequential reasoning of a clinical scientist (Supplementary Figure S1). The first layer is the molecular UP2 score, computed independently of the phenotype; the second layer is the linear regression model described above, whose fitted coefficients directly quantify the weight given to each input: the UP2 score, the PhenoGenius phenotype-matching score, and the expert-rule features (inheritance mode and trio-transmission consistency, read depth, alternate allele count, variant allele fraction, base quality and filter status).

The dataset used for training and validation was sourced between 20 October 2021 and 7 November 2023 from proprietary data. The dataset comprised 46 million variants with 678 causal variants from multiple partner genomic centers, diagnosed by certified molecular pathologists. Patients were affected by rare diseases including intellectual disability, cardiopathy and nephropathy. Causal variant was established by dual-reading by two independent pathologists. Multiple causal variant types are represented in the dataset with a majority of missense. Different configurations of analyses (trio or mono, with phenotypes or without phenotypes) were included to avoid an eventual overfitting of a particular diagnostic scenario. Variants were called by SeqOne in-house pipeline. Risk factor variants (such as APOL1 variants) were excluded. Only single-nucleotide variants (SNVs) and short indels (<300 bp) were included.

Missing numerical values were imputed to use a linear regression model. Features were imputed as 0 if the given pattern was not present or not applicable, corresponding to the biological standards of the features. Binary features already distributed between 0 and 1 were included. Concerning continuous features, UP^2^ score was rescaled using min–max normalization and PhenoGenius raw score was rescaled using mean normalization.

The dataset was split into a training set of 26 million variants, with 397 causal variants from 307 samples used to train the model parameters and a validation set of 20 million variants with 281 diagnostic variants from 252 samples used for the validation of the DiagAI score prediction.

### 2.6. DiagAI Shortlist

The prioritization of variants into a condensed shortlist is a first step in genomic interpretation, enabling the focused functional analysis of a limited set of high-probability candidates. To build a shortlist of variants most likely to be causal, two thresholds were applied: one for the molecular UP2 score and another for the DiagAI score. These thresholds were set to optimize the precision and recall. The DiagAI threshold value depends on the presence of HPO terms. A third threshold was applied to discard variants with a frequency above 1.5% in the phenotypically matched cohort. This threshold was based on the frequency, in our cohort, of the most frequent causal variant.

These thresholds were selected on the independent validation set described in Section 2.5, which is disjointed from both the training data and the nephrology evaluation cohort, by choosing the cut-offs that jointly optimized precision and recall on that held-out set; they were not tuned on the nephrology evaluation cohort.

### 2.7. Evaluation of Performance

We compared the performance of UP2 score to rank missense diagnostic variants to the following methods: CADD [31], REVEL [32] and AlphaMissense [33]. The thresholds used are taken from the original publications of AlphaMissense [33] (0.35, 0.55) and REVEL [32,34] (0.29, 0.644). We focused on missense variants, for which predictive algorithms are key to ACMG classification. To ensure the fairness of our evaluation, particularly given that our UP2 model is trained on ClinVar data, we excluded any diagnostic variants previously cataloged in ClinVar.

Three non-overlapping datasets were used in this study. UP2 was trained and internally validated exclusively on ClinVar (Section 2.3); the DiagAI integration layer was trained and validated on the separate proprietary dataset described in Section 2.5; and the 966-exome nephrology cohort was used solely for the retrospective evaluation reported here and contributed to neither training step. Because the benchmark above was restricted to causal variants absent from ClinVar, none of those variants had been seen by UP2 during training, preventing information leakage into this comparison.

To benchmark the performance of DiagAI, we compared its ranking performance to AI-MARRVEL and Exomiser (v13), which prioritizes genes or variants by leveraging information on variant frequency, predicted pathogenicity, inheritance modes, and gene–phenotype association [6,35].

Exomiser [35] was run using default pathogenicity sources MVP and REVEL, and failedVariantFilter, inheritanceFilter, frequencyFilter and pathogenicityFilter with the keepNonPathogenic option set to true. After filtering, the OmimPrioritizer and hiPhivePrioritizer steps were used. AI-MARRVEL ran with the lite version, with no access to the HGMD resource. Because DiagAI uses a filter profile based on quality, variant allele frequency, depth and number of observed alternate alleles in its scoring, we applied the same filtering prior to the run of Exomiser [35] and AI-MARRVEL. We evaluated the rankings at the gene level. For Exomiser [35], we used the gene ranks from the json output file. For both DiagAI and AI-MARRVEL, genes were ranked based on the highest-priority variant among all of their associated variants.

## 3. Results

### 3.1. Comparison Between UP2, CADD, REVEL and AlphaMissense

**Figure 1:**
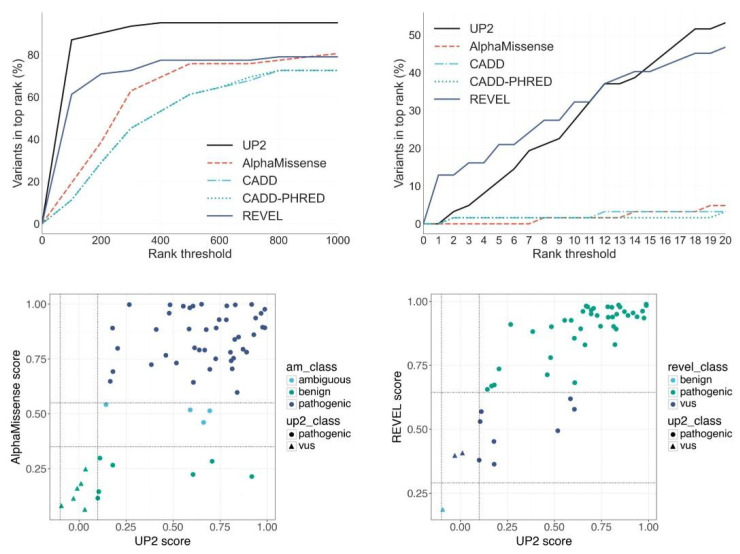
Performance of UP2 variant ranking evaluated on 62 missense variants not reported in ClinVar. (**Top-level panel**): percentage of analysis with the diagnostic variant being in the top-rank list of variants ((**left**) top 100, (**right**) top 10) according to different scoring systems: AlphaMissense, CADD, REVEL and UP2. (**Bottom-level panel**): Comparison of variants’ scores between AlphaMissense (**left**) or REVEL (**right**) and UP2 (62 causal missense variants not in ClinVar). am_class: classification predicted by AlphaMissense; up2_class: classification predicted by UP2; am_revel: classification predicted by REVEL. Dashed lines indicate the threshold of corresponding algorithms. The bottom-right corner of AlphaMissense vs. UP2 comparison corresponds to the variants not detected by AlphaMissense but reported as pathogenic by the UP2 scoring system.

The molecular component of DiagAI, UP2, scores how likely a variant is to be pathogenic. Before evaluating the full system, we tested UP2 in isolation against three widely used missense pathogenicity predictors, namely, CADD, REVEL and AlphaMissense, to ask how well each ranks the true causal variant among the candidates in a patient. We compared the ranking provided by UP2 to the ones provided by AlphaMissense, CADD and REVEL for causal missense variants from our cohort that were not reported in ClinVar (62 variants, Figure 1). Because UP2 is trained on ClinVar, restricting this test to variants absent from ClinVar ensures that the comparison is not biased in UP2’s favor. For the shorter shortlist (≤10), REVEL outperformed other tools in prioritizing causal missense variants: it ranked the causal variant first in 12.90% of cases and within the top 10 in 32.26%. In comparison, the top 1/top 10 inclusion rates were 0%/27.42% for UP2, 0%/1.61% for AlphaMissense, and 0%/1.61% for CADD. For larger variant lists (>10), UP2 outperformed other methods and ranked the causal variant within the top-100 shortlist in 87.10% of cases. In comparison, the percentages of the top-100 shortlist containing the causal missense variants were 61.29% for REVEL, 19.35% for AlphaMissense, and 11.29% for CADD.

Comparing AlphaMissense and UP2 scores only, we find strong concordance with 72.6% (45/62) of the variants classified as pathogenic by both methods. However, seven variants (11.3%) were incorrectly classified as benign by AlphaMissense, whereas they were correctly considered as pathogenic by UP2 (Figure 1, lower left panel). No variants were considered pathogenic by AlphaMissense or benign with UP2. REVEL and UP2 also showed strong concordance, with only one causal variant classified as benign by REVEL and as VUS by UP2, and no cases where one method classified a variant as pathogenic and the other as benign (Figure 1, lower right panel).

### 3.2. Interpretability of the Classifier

**Figure 2:**
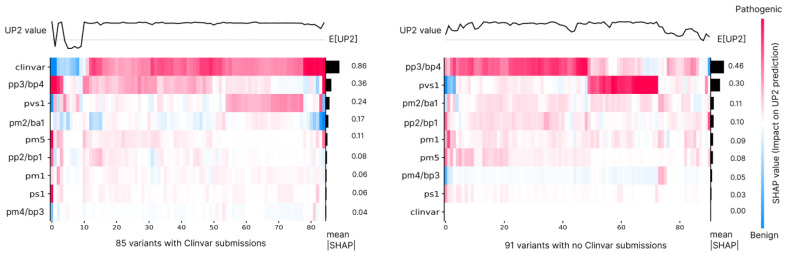
Explicability profile of the 176 diagnostic variants for the Universal Pathogenicity Predictor. The heatmap shows for each variant whether the impact of the features related to an ACMG criterion is pathogenic (red) or benign (blue). Variants that have ClinVar submissions (**left**) have UP2 predictions that are mostly influenced by the ClinVar submission predictors (number of submissions per ACMG class) with an average absolute SHAP value of 0.86 for ClinVar predictors. Variants that have no ClinVar submission (**right**) have UP2 predictions that are mostly influenced by features from PP3/BP4 or PVS1 criteria. The right-side histogram shows that the average absolute SHAP value is 0.46 for PP3/BP4 and 0.30 for PVS1 on the UP2 value for variants without ClinVar submission. The top plots show UP2 values that are between −1 and 1.

For a clinical tool, knowing why a variant received a high score matters as much as the score itself. We therefore examined which ACMG criteria most influenced UP2’s scores, using Shapley values to attribute each prediction to its underlying evidence (Methods). We applied our interpretability framework to identify the ACMG criteria most influential in determining the ACMG classifications of our cohort of 196 exomes consisting of 176 unique causal variants. Some variants were indeed shared between exomes. Among the 85 diagnostic variants with ClinVar submissions, features related to the number of pathogenic and benign submissions were the most influential (Figure 2). However, for 13 variants, other ACMG criteria were more impactful, including six with PP3/BP4 (in silico predictors of pathogenicity and benignity), five with PVS1 (predicted impact by VEP), and two with PM2/BA1 (absent or at extremely low frequency in the general population).

For the 91 diagnostic variants without ClinVar submissions, PP3/BP4 features were instrumental for 66% (60/91) of the variants, while PVS1 features were key for 29% (26/91). The scores for the remaining five variants were primarily determined by a combination of other ACMG features.

### 3.3. Proportion of Causal Variants Identified in Shortlists

A central goal of DiagAI is to compress thousands of candidate variants into a shortlist small enough for manual review while still containing the true diagnosis. We therefore asked how often the causal variant survived into the DiagAI shortlist, and how large that shortlist was, with and without the patient’s symptoms. For exomes with a confirmed molecular diagnosis, 94.9% (186/196) of causal variants were contained in the shortlist when HPO terms were used, compared to 90.8% (176/196) when HPO terms were not used. The median shortlist size was nine variants when HPO terms were not used (min = 2, max = 44) and 12 variants when HPO terms were used (min = 4, max = 29).

Although incorporating HPO terms both enlarged the shortlist (median 9 to 12 variants) and improved ranking, these effects are complementary. The shortlist is defined by a DiagAI score threshold that depends on HPO availability (Section 2.6); when phenotype terms are supplied, the PhenoGenius component raises the score of variants in phenotypically concordant genes, rescuing causal variants whose molecular score alone would have fallen below threshold (increasing recall from 90.8% to 94.9%) and, by retaining more borderline phenotype-consistent variants, modestly lengthening the list. The larger list therefore reflects higher sensitivity, whereas the improved top-rank accuracy reflects better discrimination among the retained candidates.

### 3.4. Variant Ranking

**Figure 3:**
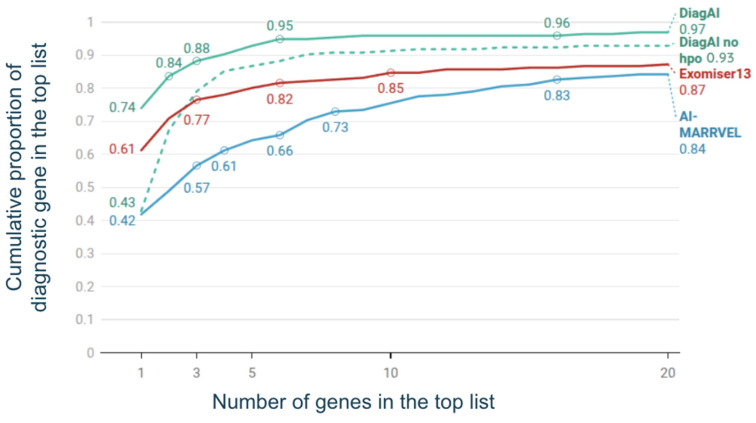
Performance of variant ranking evaluated on the 196 exomes with a confirmed molecular diagnosis. The ranking accuracy of top-ranked genes was assessed using two approaches when evaluating DiagAI: the molecular pathogenicity score alone (no HPO) and a comprehensive score integrating molecular pathogenicity and HPO-based clinical information (with HPO). Variants with a cohort frequency above 1.5% were excluded from the DiagAI ranking. Exomiser v13 and AI-MARRVEL, which also use HPO for gene ranking, were used for comparison.

Beyond whether the causal variant appears anywhere in the shortlist, its rank within that list determines how quickly a scientist reaches the diagnosis. We therefore measured how often the causal variant was the top-ranked candidate, and compared DiagAI against two established phenotype-aware prioritization tools, Exomiser (v13) and AI-MARRVEL, on the same cases. We compared DiagAI’s variant ranking accuracy with and without utilizing HPO-based clinical information (Figure 3) on the 196 exomes with a confirmed diagnostic variant. Incorporating clinical data significantly improved the ranking accuracy for the top-ranked variant. Specifically, 42% of top-ranked variants were diagnostic when HPO terms were not included, whereas this percentage increased to 74% when HPO terms were accounted for. The improvement was less pronounced when considering a larger list of 20 genes, with 93% of diagnostic variants included without HPO terms versus 97% with HPO terms. DiagAI achieved improved ranking performance compared to Exomiser v13 and AI-MARRVEL when HPO terms were provided, and for top-ranked lists of three or more variants when HPO terms were not included (Figure 3).

### 3.5. Causal Variants Absent from the Shortlists

Across the 196 diagnosed exomes, ten causal-variant calls were absent from the DiagAI shortlist; these correspond to nine distinct variants, as one variant (in CUBN) recurred in two unrelated patients. Four of the nine had been classified as benign in ClinVar (HNF1A, CFI, PODXL and ABCC6) and two as variants of uncertain significance (CUBN and NPHP3); because UP2 is trained on ClinVar labels, these received benign-leaning molecular scores that no additional evidence was available to override. A seventh variant, in COL4A4, had been classified as benign in ClinVar when the training data were assembled but has since been reclassified as pathogenic. The two remaining variants were missed for mechanism-level rather than label-level reasons: the PKD2 variant, a complex insertion, was correctly assigned a pathogenic molecular score by UP2 but was removed by the sequencing quality filter, and the NPHS2 variant lay in a recessive gene but was present as a single heterozygous call with no second qualifying variant, so no compound-heterozygous diagnosis could be assembled. The full per-variant list, with gene, genomic change, gene-level inheritance, ClinVar classification at the time of analysis, UP2 molecular (ACMG) class and the reason each was missed, is provided in Supplementary Table S2.

## 4. Discussion

Our study demonstrates DiagAI’s effectiveness in prioritizing causal variants in exomes from nephrology patients, analyzed as single cases rather than trios. Depending on the availability of phenotypic data, 90.8 to 94.9% of shortlists contained the causal variant. DiagAI also outperformed Exomiser v13 and AI-MARRVEL in gene-level ranking comparisons.

We also compared UP2 to widely used variant scoring tools to assess its utility in clinical variant prioritization, focusing specifically on causal missense variants not reported in ClinVar. REVEL performed well in ranking top variants, but failed to capture several true positives as shortlist size increased. In contrast, UP2 maintained strong performance across broader ranking ranges, making it more suitable for diagnostic applications where sensitivity is essential. AlphaMissense and CADD showed consistently lower performance, underscoring the added value of UP2 and, to a lesser extent, REVEL for effective variant prioritization.

DiagAI’s outperformance of open-source alternatives is explained by several key features. First, the UP2 algorithm was meticulously designed through the selection and analysis of a comprehensive set of features to enhance the model’s ability to identify pathogenic variants. A key contribution also lies in the rigorous data curation process during the ClinVar data treatment, addressing conflicting ClinVar classifications [20]. Furthermore, we implement data augmentation techniques to mitigate biases, particularly those stemming from ClinVar’s inherent skew toward pathogenic variants, which can distort model performance, for example, in intergenic regions. Secondly, DiagAI implements a modular two-layer architecture that integrates machine learning models trained on patient cohorts with expert-driven methodologies to capture complex patterns. This approach addresses critical challenges, including the accurate assessment of variant inheritance, notably the interpretation of compound heterozygous variants, as well as the evaluation of data quality and the management of sequencing technology differences. The integrated design not only strengthens diagnostic reliability, but also enhances the framework’s adaptability to a wide range of data types, including long-read sequencing (in a small exploratory set of ten long-read whole-genome cases, DiagAI retrieved the causal variant in every case; given the very limited sample size this is presented only as a preliminary observation of feasibility).

Several of the variants missed by DiagAI reveal intrinsic limitations of the approach. The *COL4A4* case illustrates a fundamental limitation of any ClinVar-trained classifier: its ceiling is set by the state of the reference database at training time, so a variant that was benign in ClinVar when the model was trained but is pathogenic today can be recovered only by retraining on current ClinVar. The *PKD2* case shows that the sequencing quality gate, although necessary to control false positives, can also discard a genuine call. The remaining variants, *PODXL*, a recently characterized gene–disease association [36]; *CFI*, an incompletely penetrant complement variant that is difficult to classify under ACMG criteria [37] and is often treated as a risk factor rather than causative in Mendelian disease [38]; and *NPHP3*, a synonymous variant with a cryptic splicing effect not captured by in silico predictors [39], illustrate the categories of variant that remain beyond the reach of current pathogenicity models and continue to require expert review.

These cases illustrate the inherent challenges in variant interpretation, particularly for variants with emerging or complex pathogenic mechanisms that are not yet well captured by computational models. While DiagAI improves variant prioritization by leveraging multivariate ACMG evidence tags and machine learning trained on ClinVar ACMG classifications, it remains limited by the available knowledge and data used for training. By integrating diverse evidence sources and phenotypic data encoded with HPO terms, DiagAI enhances variant ranking, but expert review remains essential for capturing novel or particularly challenging cases.

DiagAI contributes to the growing landscape of computational solutions for variant prioritization, joining both open-source and commercial offerings such as AI-MARRVEL, Invitae MOON, Fabric GEM, and the Emedgene v100.40.0 software from Illumina [6,8,9,40]. Direct comparisons between these tools are challenging due to differences in their evaluation cohorts. Nonetheless, we found that DiagAI outperformed AI-MARRVEL in gene ranking and demonstrated comparable performance in identifying diagnosable cases, with both tools automatically detecting 50–60% of such cases [6]. For a fair comparison, the Critical Assessment of Genome Interpretation (CAGI) challenge offers a standardized benchmarking framework; however, DiagAI has yet to be evaluated within this context.

Several limitations frame these results. Although the DiagAI integration layer was trained on a cohort spanning intellectual disability, cardiopathy and nephropathy, the evaluation reported here was restricted to adult nephropathy. Prospective, specialty-specific validation—for example, in pediatric, neurodevelopmental and cardiology cohorts—will be required to establish generalizability across clinical specialties. The missense benchmark, restricted to causal variants absent from ClinVar, was limited to 62 variants, which widens the confidence interval around the reported rates (for the UP2 top-100 inclusion rate of 87.10%, the 95% Wilson score confidence interval is 76.6–93.3%); this benchmark will be expanded using more recent ClinVar releases and additional curated external sources. Finally, although the upstream GermlineVar pipeline detects structural variants with high sensitivity using GRIDSS and AluMEI, the current DiagAI model prioritizes only single-nucleotide variants and short indels (<300 bp); structural variant prioritization is a planned extension of the framework.

DiagAI’s accuracy in variant ranking, particularly when integrating clinical data, highlights its potential to streamline genomic diagnostics by reducing the number of variants requiring manual review. However, the path to full automation remains long, with less than 60% of diagnosed cases detected automatically. These findings suggest that AI-powered tools like DiagAI can significantly reduce the interpretive workload in clinical genomics while maintaining high diagnostic accuracy. This assessment should be evaluated beyond the specific case of nephrology and further tested in the context of whole-genome sequencing analysis.

## Data Availability

DiagAI and its UP2 component are proprietary clinical software developed by SeqOne Genomics and are offered as part of the company’s commercial diagnostic platform; the underlying model code and training data are therefore not publicly distributed. The PhenoGenius phenotype-matching algorithm is open source and available on GitHub (https://github.com/kyauy/PhenoGenius (accessed on 17 December 2024)), as is the ClinVCF curation tool used to prepare the training data (https://github.com/SeqOne/clinvcf (accessed on 17 December 2024)). To allow readers and reviewers to assess the results independently of access to the proprietary software, the benchmark evaluation data—the ranked candidate lists produced by each compared tool for the causal variants analyzed here—together with the scripts used to compute the reported metrics are available upon request. The patient-level sequencing data underlying this study are subject to consent and privacy restrictions and are available from the corresponding author (L. Mesnard) on reasonable request, subject to the applicable ethical and data protection approvals.

## Supplementary Materials

The following supporting information can be downloaded at: https://www.mdpi.com/article/10.3390/cimb48070706/s1.

## Abbreviations

The following abbreviations are used in this manuscript:

ACMG	American College of Medical Genetics and Genomics
AI	Artificial intelligence
ES	Exome sequencing
HPO	Human Phenotype Ontology
SNV	Single-nucleotide variant
UP2	Universal Pathogenicity Predictor
VUS	Variant of uncertain significance
WGS	Whole-genome sequencing
