# Supplementary material for: Integration of Machine Learning-Based Pathogenicity Prediction and Phenotype Matching Improves Variant Prioritization in Rare Clinical Testing
Source: Curr Issues Mol Biol. 2026 Jul 11;48(7):706. doi: 10.3390/cimb48070706 (PMC13406839; doi:10.3390/cimb48070706)
Supplement: Supplementary file 1 [file cimb-48-00706-s001.zip › cimb-4392360-supplementary.pdf]

Supplemental Table S1. Characteristics of the patients included in the nephrology cohort used to evaluate DiagAI. For continuous variables, we provide mean and standard deviation in parentheses. For discrete ones, we provide counts and proportions in parentheses. CKD: Chronic Kidney Disease, ES: Exome Sequencing.

Characteristic	Overall N = 966
Age, years	45 (15)
Gender, male	573 (69%)
Self-declared ancestry	
Europe	344 (36%)
North Africa	165 (17%)
Sub-Saharan Africa	277 (29%)
Asian	44 (5%)
Others or unknown	136 (14%)
Diagnosed with exome, positive	196 (20%)
Kidney biopsy performed	346 (36%)
First-degree history of CKD	311 (32%)
Mother	99 (10%)
Father	87 (9%)
Brother	99 (10%)
Sister	69 (7%)
Child	27 (3%)
First or second-degree history of CKD	409 (42%)
CKD stage at the time of ES	
1	137 (16%)
2	99 (11%)
3	205 (24%)
4	133 (15%)
5	229 (26%)
Unknown	63 (7%)

Supplementary Table S2. False negative cases. The nine distinct variants underlying the ten false-negative cases, with gene, genomic change (GRCh37), number of patients in which the variant was observed, gene-level inheritance, ClinVar classification at the time of analysis, UP2 molecular (ACMG) class and the reason each was missed.

Gene	Variant (GRCh37)	Patients	Inheritance	ClinVar at analysis	UP2 ACMG class	Why it was missed
HNF1A	chr12:121,434,074 A>G	1	AD / AR	Benign	**2.5**	ClinVar-benign at analysis; benign-leaning UP2 score, no evidence to override
CFI	chr4:110,667,561 T>G	1	AD / AR	Benign	**1**	ClinVar-benign; incompletely penetrant, frequently treated as a risk factor
PODXL	chr7:131,196,015 G>C	1	AD / AR	Benign	**2**	ClinVar-benign; gene–disease association established only recently

Gene	Variant (GRCh37)	Patients	Inheritance	ClinVar at analysis	UP2 ACMG class	Why it was missed
ABCC6	chr16:16,272,764 C>T	1	AD / AR	Benign	**1**	ClinVar-benign; benign UP2 score
CUBN	chr10:16,970,304 T>C	**2**	AR	VUS	**2.5**	ClinVar-VUS; insufficient evidence to score above threshold (observed in two unrelated patients)
NPHP3	chr3:132,415,592 G>A	1	AR	VUS	**2**	ClinVar-VUS; synonymous variant with a cryptic splicing effect not modelled in silico
COL4A4	chr2:227,984,645 C>T	1	AD / AR	Benign at analysis → since reclassified Pathogenic	**2.5**	Trained on the then-current ClinVar (benign); the classification itself has since changed
PKD2	chr4:88,929,301 15-bp insertion	1	AD / AR	,	**5**	Correctly scored pathogenic by UP2 but removed by the sequencing-quality filter (complex insertion)
NPHS2	chr1:179,520,385 C>T	1	AR	,	**3.5**	Recessive gene present as a single heterozygous call; no second qualifying variant for a compound-heterozygous diagnosis

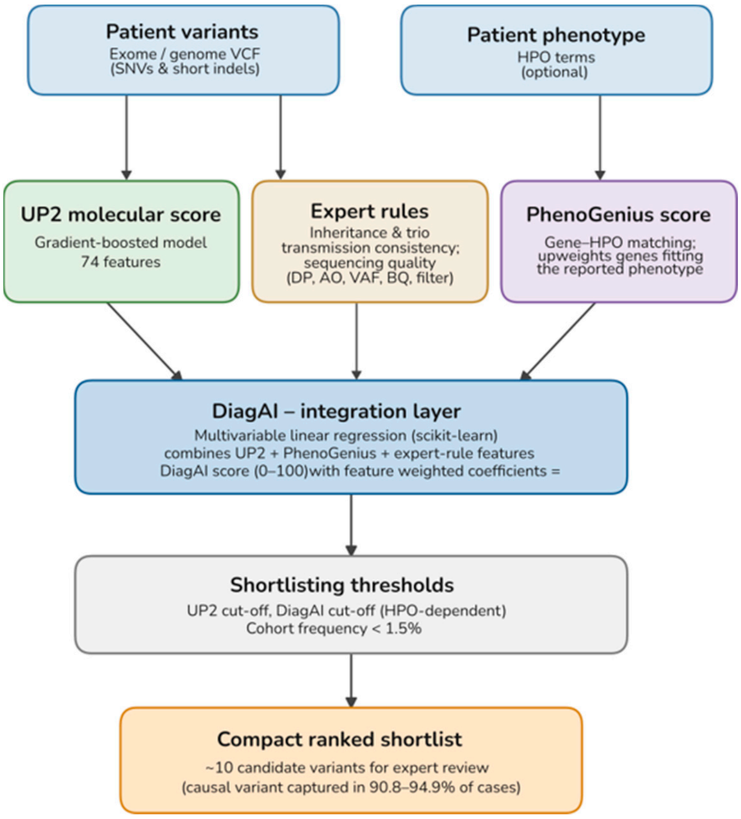

Supplementary Figure S1. Overview of the DiagAI variant-prioritisation framework. Candidate variants from exome or genome sequencing and, when available, the patient's HPO terms enter a two-layer model. The first layer is the molecular Universal Pathogenicity Predictor (UP2). The second layer is a linear regression that combines the UP2 score, the PhenoGenius phenotype-matching score and expert rules (inheritance-mode and trio-transmission consistency, and sequencing quality) into a single DiagAI score (0–100). Score thresholds ,whose DiagAI cut-off depends on HPO availability ,and a cohort-frequency filter (<1.5%) then produce the final compact ranked shortlist for expert review.
